# Hyperspectral Imaging for High Throughput Optical Spectroscopy of pL Droplets

**DOI:** 10.1021/acs.analchem.4c04731

**Published:** 2025-01-29

**Authors:** Marc Sulliger, Jaime Ortega Arroyo, Romain Quidant

**Affiliations:** Nanophotonic Systems Laboratory, Department of Mechanical and Process Engineering, ETH Zurich, 8092 Zurich, Switzerland

## Abstract

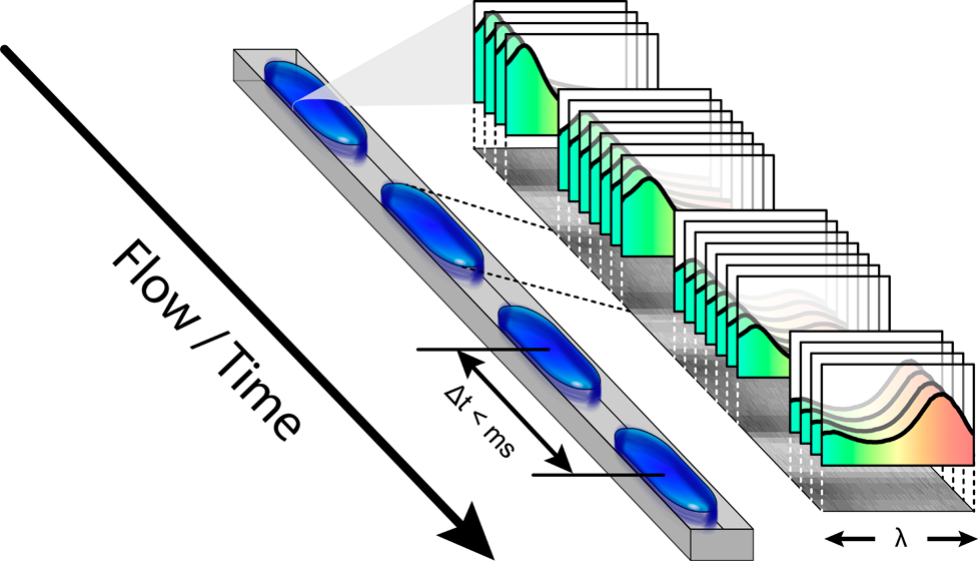

Droplet-based microfluidics is a powerful tool for high-throughput analysis of liquid samples with significant applications in biomedicine and biochemistry. Nevertheless, extracting content-rich information from single picolitre-sized droplets at high throughputs remains challenging due to the weak signals associated with these small volumes. Overcoming this limitation would be transformative for fields that rely on high-throughput screening, enabling broader multiparametric analysis. Here we present an integrated optofluidic platform that addresses this critical point by combining advanced hyperspectral imaging with self-referencing and measurement automation. With this approach our platform achieves high temporal and spectral resolution with shot-noise limited performance, allowing for the label-free interrogation of single droplet contents. To demonstrate the platform’s capabilities, we first exploit its high temporal and spectral resolution to study rapid dynamic changes in the composition of a heterogeneous population of nanoparticles. Second, leveraging the platform’s shot-noise limited performance and using a model DNA-AuNP sensor, we detect target DNA sequences down to 250 pM, thereby showcasing the platform’s compatibility with demanding sensing applications. Finally, through measurement automation, we demonstrate multiplexed sample monitoring over hours. These findings show that our optofluidic platform not only helps to close the current gap in high-throughput droplet analysis, but also significantly advances the potential for content-rich characterization, ultimately enhancing the scope and effectiveness of high-throughput screening methods.

State-of-the-art microfluidic devices are nowadays routinely applied to a broad range of samples–be it in the context of medicine,^1−3^ chemical synthesis,^4,5^ biochemistry,^6,7^ or material science.^8,9^ A prominent example of this is droplet-based microfluidics. The benefits of droplet-based microfluidics combined with optical readout have been shown extensively in many research articles and review papers.^10−13^ In terms of experimental procedure, droplets themselves define individual but identical reaction containers making each droplet an independent experiment. Therefore, droplets allow for high throughput measurements—where high throughput is defined as the number of experiments performed per unit time—and only require a small volume. In sensing applications, each droplet acts as an individual 3D sensing volume/assay. When in motion, internal flow fields facilitate continuous sample circulation and mixing. Moreover, microfluidic droplet chips offer a diverse toolbox of design elements enabling complex on-chip functionalities. Additionally, the “closed” nature of droplets prevents contamination of the chip’s region of interest (ROI), facilitating chip reusability and reducing production cost.

Typical methods to analyze droplets can be categorized into two main approaches. The first is to perform a digital assay, hence assigning a binary value to each droplet, e.g. whether a certain species is present or not, and subsequently accumulating large numbers of droplets to determine the underlying concentration of a target analyte with high sensitivity.^14^ Digital assays often require off-chip incubation as well as dedicated read-out chips. More importantly digital approaches generally do not provide time-resolved information and depend on the stochastic loading of each droplet (i.e., <1 target per droplet). The second approach consists of assigning analog values to each droplet by either spectroscopic or electrochemical techniques. Despite enabling time-resolved or high temporal resolution studies, applications based on analog signal processing predominantly compare a single measurement value (i.e., single wavelength absorbance/emission) to a threshold, therefore, binarizing the information about each droplet’s content. To increase the information content, broadband spectral droplet read-out has been proposed for different spectroscopic techniques.^15−20^ While some of these approaches achieve considerable sensitivity, they are all limited either by the data throughput (spectra/s, minimum sample volume, temporal resolution), the complexity in the instrumentation (e.g., cavity enhancement), or the requirements for the chip fabrication (e.g., chip integrated optical fibers or optical path length enhancing geometries).

Hyperspectral imaging (HSI),^21^ initially developed for earth remote sensing,^22^ is a promising approach to address the throughput limitations of current broadband spectral readout schemes. HSI simultaneously records spatial and spectral data over a large field of view (i.e., allows position dependent time encoding), resulting in several hundred spectra in a single image. Mekki-Berrada et al.^23^ recently applied HSI to interrogate mm-sized, plug-like droplets at low throughputs (approximately 26 Hz) in a nonmicrofluidic context. While such work demonstrates that HSI can be applied to moving liquids, the more than 10′000-fold droplet volume and 100-fold throughput mismatch poses significant challenges for extending such an approach to high throughput screening applications ideally suited to droplet microfluidics,^24^ such as detection of disease biomarkers,^25^ single cell genetic profiling,^26^ and drug^27^ or enzyme discovery.^28^

Unfortunately, bridging said gap is not as straightforward as simply reducing the droplet volume, since this not only significantly decreases the optical path lengths (OPL) and in turn drastically lowers the signal-to-noise ratio (SNR), but also places restrictions regarding material choices, sample delivery techniques and liquid manipulation. Furthermore, due to the decrease in the observable signal magnitudes, minute background fluctuations, once considered negligible, may now severely affect the experiments, and thus measurement stabilization techniques covering short- and long-term influences are required.

In this work we aim at overcoming all these limitations by combining different tools into an integrated optofluidic platform. To achieve small volumes and high throughput we use state-of-the art droplet-based microfluidic polydimethylsiloxane (PDMS) chips with integrated push-up valves. For optical broadband interrogation we apply HSI in a push-broom implementation in order to compensate for low OPL by detecting hundreds of spectra in a single shot. Next, to stabilize measurements against any kind of fluctuations we implement a two-step self-referencing approach that corrects for drifts even over extended (hours) periods of time. Finally, to acquire precise and reproducible data we automate all essential components of the assay, allowing experiments to run fully independent of user inputs once started.

We here present and characterize a microfluidic droplet chip along with a modular optical HSI setup in combination with a readout workflow including advanced self-referencing for fluctuation/drift correction. To showcase the potential of our platform to interrogate pL volumes with high temporal and spectral resolution, we resolve fast dynamical changes in sample composition. Additionally, using a model DNA sensor comprising nucleic acid functionalized gold nanoparticles (AuNPs), we demonstrate two potential applications of our platform: high throughput biosensing and real-time monitoring of complex samples.

## Materials and Methods

### Droplet Chip Characterization

The chip (see Figure 2b and Supporting Information for dimensions) was characterized by producing (Milli-Q) water droplets under different oil and aqueous phase pressure conditions, respectively. Specifically, the oil phase pressure was scanned from 3300 to 500 mbar in 100 mbar steps, while keeping the aqueous phase pressure fixed. The above oil phase scan was repeated for aqueous phase pressures from 2400 mbar down to 500 mbar in 100 mbar steps. For each condition, 1000 frames were recorded over 2 s. The droplet frequency and size were then determined from all the droplets recorded.

### Noise/Sensitivity Assessment

For noise assessment, a total of 55,775 droplets containing 40 nm AuNPs (droplet frequency = 1030 Hz, length = 86.5 ± 1.7 μm) were recorded and analyzed. From this data set, all droplets detected in the center region of the ROI (length = 104.1 μm, 6056 droplets, 30 spectra per droplet) were further processed and presented in Figure 4d. After averaging a certain number of spectra (i.e., droplets), the average and standard deviation (STD) of the absorbance were determined for each wavelength. The average value of the STD for the spectral region of 520–550 nm was then used as a metric for the noise.

### Experimental Procedure and Data Analysis for Fast Dynamical Changes

Stock solutions of 40 and 80 nm spherical AuNPs and nanorods (approximately 5-fold concentrated by centrifugation) were used as samples. The sequence of valve actuations was fully automated, and their values stored as ground truth, whereby each valve state was active for 500 ms, except for the first and last one due to manual camera triggering. For droplet imaging, the HSI module was run at 500 frames per second and 20 individual spectra were considered per droplet to increase SNR. For Figure 5c–e, all droplets within a frame were averaged to a single spectrum. For the droplet library presented in Figure 5b, each sample was recorded independently during 2 s and all recorded droplets were averaged to a single spectrum.

### DNA-AuNP Preparation and DNA Sensing

AuNPs were functionalized with DNA according to a microwave assisted heating-drying protocol.^29^ In short, equal volumes of 40 nm AuNP at 2.2 nM and DNA at 10 μM were mixed in a small glass vial. The DNA component comprised a mixture of short DNA doped with a percentage of either probe strand A or B. The complete volume of this mixture was typically kept below 120 μL to facilitate evaporation. The glass vial was then placed in a microwave oven at a power of 1000 W until the liquid was completely evaporated–typically around 20 min. Afterward, the AuNPs were resuspended in 300 μL nuclease free water and transferred to a 500 μL DNA LoBind Eppendorf tube (0030108035, Eppendorf, Switzerland) and washed three times by centrifugation (4700 rpm or 1400*g*, mySPIN 12 mini centrifuge, Thermo Fisher Scientific, Switzerland). Before use, the concentration of each particle and doping type was equilibrated to a common starting value. Finally, both probe strand functionalized AuNPs were mixed with either target or control DNA (in 1.5× TAE with 1 M NaCl) in a 1:1:2 v/v/v ratio and incubated for at least 60 min at room temperature for end-point measurements. Acquisition of all droplet data was fully automated whereby each sample was sequentially measured (1000 frames over 2 s; 35 μs exposure time) from highest to lowest concentration. The production frequency reached up to 2.1 kHz. All recorded droplets were averaged to generate a single spectrum. Flow cell data were recorded over 5 s with a frame rate of 100 Hz. All recorded HSI frames were temporally averaged and referenced to a background measurement. At nine equally spaced positions along the spatial axis, 30 spectra were read-out and averaged to a single spectrum. To quantify the degree of agglomeration, the absorbance ratio λ_peak_/λ_610 nm_ was chosen as a metric for all data sets, and, for flow cell data, fit to a four parameter logistic regression (4PL, see Supporting Information) model, whereby, the *R*^2^ values were found to be 0.994, 0.996, 0.995, and 0.998 for 40, 30, 20 and 10% probe strand doping.

### Reaction Monitoring

The samples described above were loaded into the droplet chip immediately after mixing and measured thereafter. Acquisition of all droplet data was fully automated, whereby each sample was measured sequentially (1000 frames over 2 s; 35 μs exposure time) from highest to lowest concentration every 5 min. The first measurement cycle started 6.5 min after addition of the target strand, corresponding to the dead time associated with the preparation of all the samples and triggering the automated acquisition. Droplet length was below 70 μm, while the production frequency reached up to 1.5 kHz. All recorded droplets for a given time point were averaged to generate a single spectrum.

## Results and Discussion

### Working Principle of the Platform

**Figure 1:**
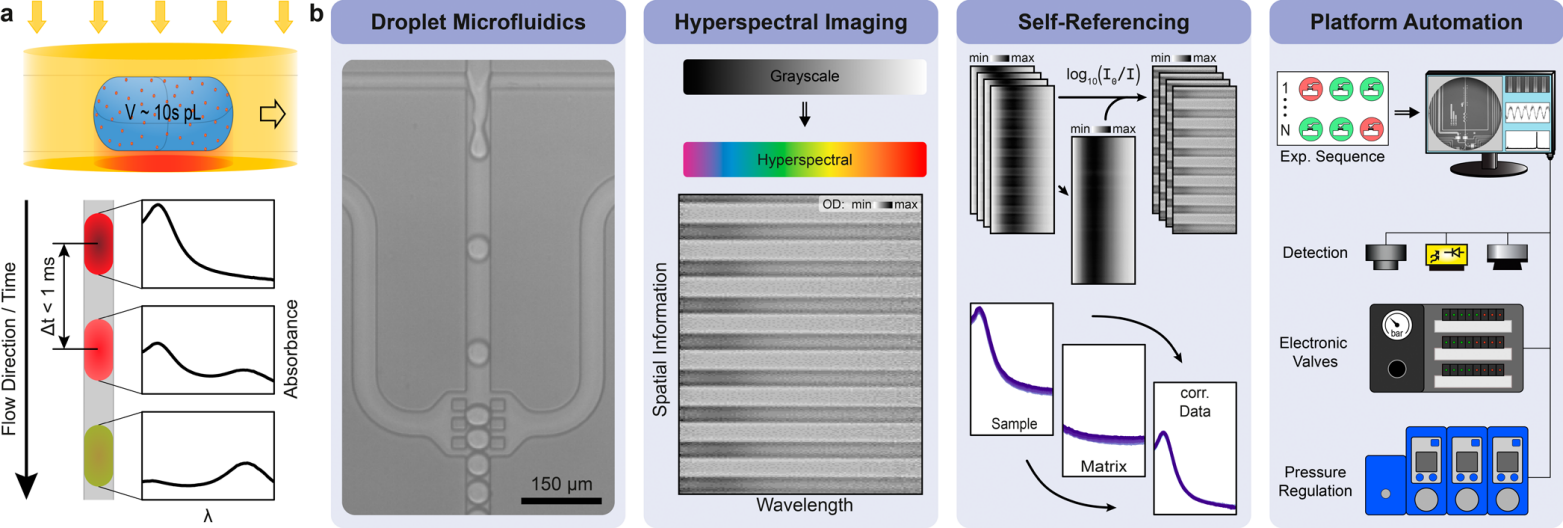
Concept and main building blocks of the experimental platform. (a) Conceptual illustration of the platform. (b) The platform comprises four main building blocks: a droplet microfluidics toolbox integrated with micro valves, a custom-built microscope for label free HSI with high spectral resolution, a self-referencing read-out approach for various (fluctuation/drift) corrections, and an automated measurement and droplet analysis workflow. All plots and images correspond to experimental data.

This work addresses the need for content-rich information with high spectral and temporal resolution at the single droplet level via broad-band absorbance spectroscopy by developing an integrated optofluidic platform based on four key components depicted in Figure 1: microfluidics, optical imaging, self-referencing, and automation.

For microfluidics, the platform takes advantage of the high throughput and low sample volume consumption of droplet-based microfluidics. In addition, we integrate valves into the microfluidic chip to further increase control over the sample and to also deliver multiplexed sample readout. For optical imaging, the platform consists of a custom microscope with multiple channel readouts, with HSI as the main detection channel. The HSI approach simultaneously extracts spatial and spectral information within a single measurement. Compared to confocal analogs, HSI delivers higher information content per read-out, as each row of the sensor records a spectrum of the sample. Recording several spectra per frame effectively collects more photons, which for shot-noise limited systems results in a higher SNR and hence a higher sensitivity. Regarding self-referencing, our technique relies on an internal and an external self-referencing step to intrinsically and reliably correct for various influences (including short- and long-term drifts such as focus position, static imprints, channel defects, channel position and alignment, pressure fluctuations, etc.), thereby stabilizing the readout over several hours and hence enabling the detection of weak sample signals. Finally, for automation, the entire platform including pressure controllers, electronic valve actuation, microscope control and data acquisition are programmatically operated and synchronized. This not only enables complex experimental protocols, but also allows for observation of very short, exactly timed sequences as well as long-term monitoring (hours) of slow dynamical changes within a specific sample.

### Microvalves Integration Delivers Enhanced Control and Multiplexing to Droplet Microfluidics

**Figure 2:**
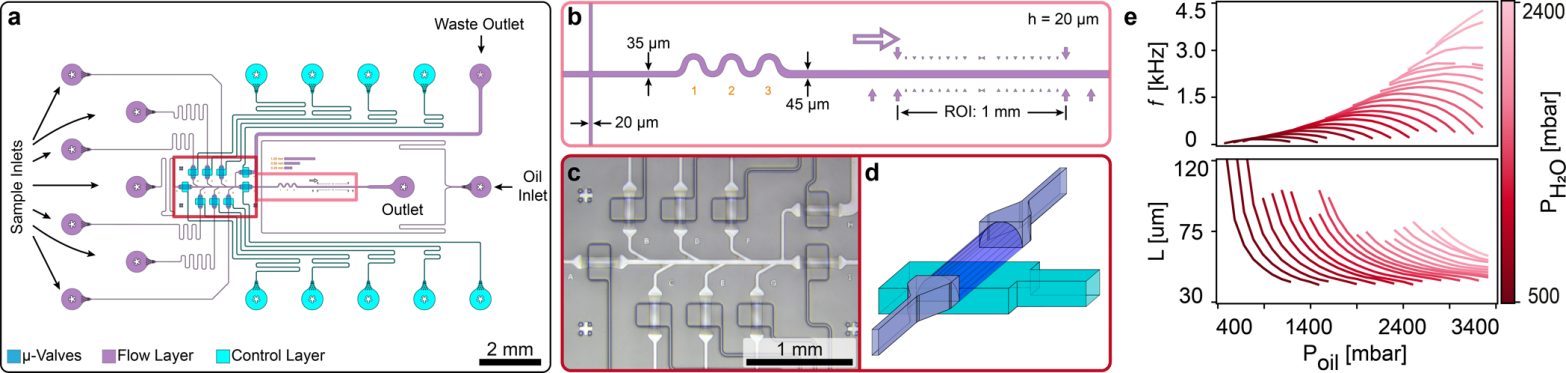
Design and characterization of droplet-based microfluidic chip. (a) Design overview. (b) Zoom onto outlet channel and ROI. (c) Image of the sample inlet/valve region of an assembled droplet microfluidic chip. (d) Schematic representation of a push-up valve where the control channel is located beneath the flow channel. (e) Characterization of droplet generation in terms of droplet production frequency and droplet length as a function of the oil pressure.

Our microfluidic chip (see Figures 2a and S1) combines droplet production and read-out within the same device. The chips are based on a two-layer architecture with microfluidic Quake valves^30^ in a push up configuration.^31,32^ We integrated microvalves into the chip with the specific aim to increase the operational control of the chip by enabling multiplexed, fully automated operation of the sample inlets, controlled sample mixing, and suppression of reagent counterflow. Push-up valves were preferred over traditional push-down valves for two reasons. First, push-up valves close low aspect ratio channels at lower applied pressures than push-down ones.^31^ Second, placing the control layer beneath the flow channel creates a uniform channel surface, entirely made of PDMS, which eliminates the need for hydrophobic surface treatment of the flow channel.

To test the versatility of the microfluidic chip, we characterized the droplet length and production frequency as a function of the only two tunable parameters during an experiment: the oil and aqueous phase pressures. To do so we scanned the pressure of the oil phase while keeping the pressure of the aqueous phase fixed; and repeated this over a range of aqueous phase pressures (Figure 2e). The parameter space sweep shows droplet length and production frequency tuneability from 45 μm to more than 100 μm and 100 Hz to 4.5 kHz, respectively. This corresponds to droplet volumes between 40.5 and 90 pL (for a droplet size of 100 μm) and flow rates between 0.25 μL/min and more than 24.3 μL/min. Ultimately, the chip’s channel geometry and dimensions determined the overall range of the droplet parameters. For most experiments reported hereafter, we targeted the production of 70 μm long droplets at a rate of 1–2 kHz (corresponding to droplet volumes of 63 pL and a sample flow rate of 3.8 to 7.6 μL/min).

### Real-Time Droplet Monitoring with a Multichannel HSI Microscope

**Figure 3:**
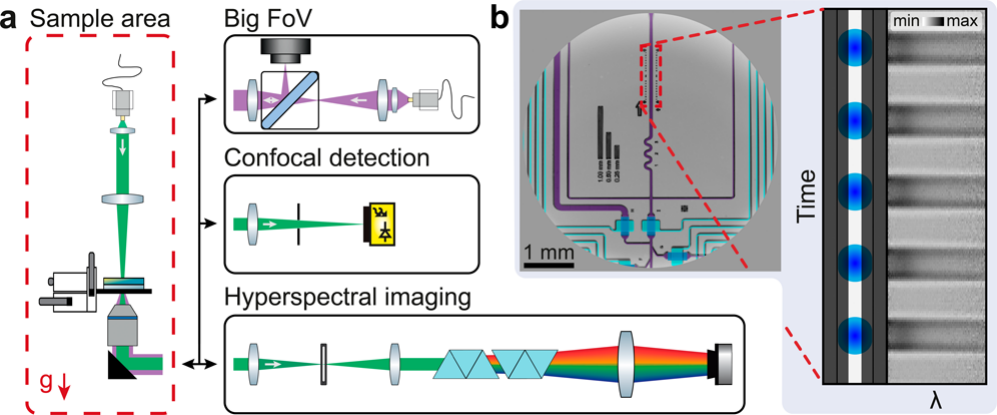
Multichannel HSI for real-time droplet monitoring. (a) Schematic diagram of the optical setup composed of: sample area (g = direction of gravity), big FoV, confocal detection, and HSI path. (b) Representative big FoV image of a microfluidic chip with false-color channels (light blue = control channels, blue = microvalves, purple = flow channels). Inset: Schematic representation of the ROI as observed through a slit and corresponding hyperspectral image (of actual droplet data) after dispersion of light perpendicular to the slit and postprocessing.

Figure 3a shows the custom-built multichannel optical setup based around a push-broom^33^ implementation of a transmission HSI-microscope, allowing us to record up to 448′000 spectra/s. The optical setup comprises three main detection modules, corresponding to an HSI, a big field of view (FoV), and a confocal-detection path. Along the HSI path, an adjustable slit in the conjugate image plane sections the illuminated FoV to a quasi-1D line, which is subsequently dispersed perpendicular to the slit by two consecutive Amici prisms. The Amici prisms play two significant roles. First, they reduce optical aberrations, and the common distortions associated with HSI such as “smile” and “keystone”.^34^ Second, they enable fine-tuning of the spectral dispersion by simply adjusting the relative prism angle with respect to the optical axis.^35^

The big FoV channel (Figure 3b) is based on a reflection implementation of a partially coherent inline holographic microscope aimed at providing an overview of the microfluidic chip. Specifically, this channel monitors chip operation like valve actuation, droplet formation, defects, and positioning of the sensing area with respect to the slit. Finally, the confocal detection module uses a pinhole together with a Si-based avalanche photodetector (APD) to monitor droplet production frequency by recording light intensity time-traces.

### Robustness and Long-Term Stability Based on Self-Referencing

Despite the many advantages of droplet-based microfluidics, challenges remain in their analysis, particularly due to droplet-specific artifacts caused by the curved surfaces and the refractive index mismatch between immiscible phases. These artifacts mainly distort optical measurements, posing a significant barrier to reliable, sensitive, and reproducible long-term studies. To mitigate these artifacts, we implemented both hardware- and data processing-based solutions. On the hardware side (Figure S2), we used refractive index matching, plug-like shaped droplets within rectangular channel cross sections; and precise channel-slit alignment. Refractive index matching achieved by doping the oil phase with 1,3-bis(trifluoromethyl)-5-bromobenzene,^15,36^ reduces scattering contributions and spherical aberrations induced by the droplet/oil interface. The combination of rectangular cross-section and plug-like droplets confines the curved parts of the droplet to the leading and trailing fronts, ensuring a uniform OPL for the central region of the droplet, which comprises approximately 75% of its length. Whereas channel-slit alignment ensures consistent measurements across the spatial axis in the HSI channel.

**Figure 4:**
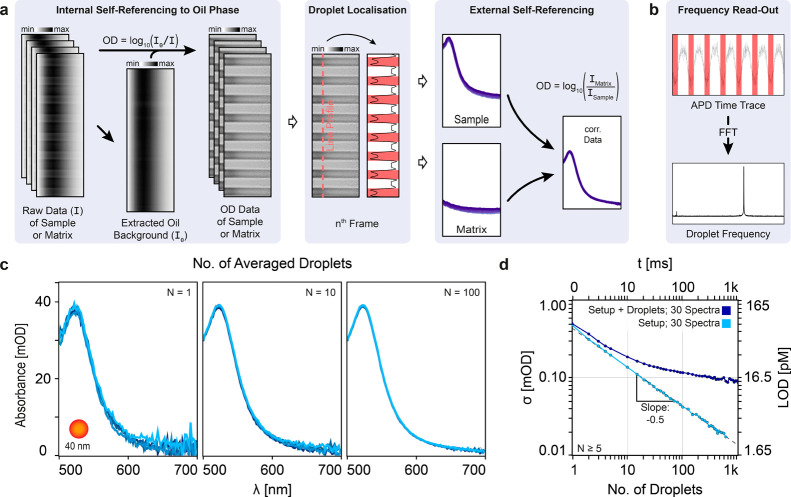
Data analysis workflow and spectral noise assessment. (a) Schematic description of the data analysis pipeline. In short: OD data are extracted by internal self-referencing to an extracted oil background. The droplets themselves are then localized by means of a line-profile along the spatial axis of the hyperspectral image. Finally, an external self-referencing step accounts for matrix contributions to observe the correct spectra. Note that all images and plots correspond to data from real droplet measurements. (b) The FFT of the APD time trace provides the production frequency of the droplets. (c) Extracted spectra of 40 nm AuNPs as a function of number of droplets averaged (*N* = 1, 10, or 100 droplets), demonstrating the increase in SNR. Each colored line represents an independent average of droplet spectra. (d) Average noise in the spectral region of 520–550 nm as a function of the number of averaged droplets (bottom axis), and corresponding effective acquisition time (top axis) for a production frequency of 1030 Hz. The right *y*-axis represents the corresponding LOD (3σ) for droplets loaded with 40 nm AuNPs.

In addition to the hardware-based solutions, we introduced two self-referencing steps, referred here as internal and external, into the data analysis pipeline outlined in Figures 4 and S3. The data processing pipeline starts with a stack of hyperspectral images (HSI stack), which have been previously mapped to the wavelength reference system (see Supporting Information and Figure S4), followed by internal self-referencing, then droplet localization and spectra retrieval, and finally external self-referencing. The internal self-referencing involves generating an optical density (OD) data set based on Beer–Lambert law, using the oil background extracted from each HSI acquisition as reference (Figure S5). Importantly, this step corrects for artifacts that remain constant within the acquisition time of the HSI stack, such as channel defects, focus position, channel position, and channel-slit alignment. External self-referencing involves subtracting matrix-only droplet spectra from the sample spectra, which requires the acquisition of a separate matrix-only HSI stack. This step corrects for matrix effects, but also accounts for artifacts associated with long-term changes (time scales longer than the acquisition of a sample and matrix-only HSI stacks) in system parameters such as light intensity and spectral drift, pressure fluctuations and drifts in sample position. This is possible, because the matrix-only spectra is internally self-referencing as well.

We validated the accuracy and long-term stability of our platform and data processing pipeline against standard approaches, showing excellent agreement with pL droplet spectra (Figure S6). Furthermore, we demonstrate its robustness over more than 4 h of continuous operation (Figure S7).

In addition to spectral information obtained from the HSI channel, our platform simultaneously records an APD time trace from the confocal-based detection channel (Figure 3a), from which the droplet production frequency is determined via Fourier transform analysis (Figure 4b).

### Spectral Sensitivity

The sensitivity of the platform was assessed with the spectra retrieved from individual droplets loaded with 40 nm spherical AuNP (Figure 4c). For this we used two complementary metrics, the first one evaluates the contribution from all noise sources (dark blue, Figure 4d), whereas the other only considers those intrinsic to the optical system (light blue, Figure 4d). For the latter, we calculated the spectral fluctuations between two successive droplets, a differential measurement, as a function of number of droplets averaged. As shown in Figure 4c,d, these data show the characteristic scaling associated with shot-noise limited performance. A noise assessment over the full spatial extent of the ROI as well as the entire spectral window is shown in Figure S8.

The curve including all noise sources, however, deviates from ideal shot-noise limited performance. This deviation arises from the limitations of the self-referencing approach; namely, only changes (drifts/fluctuations of focus position, light source, pressure) slower than the time it takes to acquire an internal and external self-reference are fully corrected for. Nevertheless, for an average of only 10 droplets the sensitivity reaches 0.184 mOD, dropping to 0.116 mOD for an average of 100 droplets. Compared to Probst et al.^15^ (0.13 mOD, averaged over 500 droplets recorded during 11.11 s), we achieve the same sensitivity approximately 275-fold faster, with only 40 droplets averaged, and with 20% shorter OPL. Based on the effective noise limit of the platform, one can now calculate the limit of detection (LOD, 3σ) for any system with a spectral output in the given wavelength window. For instance, for droplets containing 40 nm AuNPs, observed in a channel with 20 μm height, averaging 100 droplets leads to a LOD of 19.1 pM. Considering that each individual spectrum samples a volume of about 0.5 pL, the detection limit for 40 nm AuNPs equals to 6 particles/spectra.

While the self-referencing approach presented here is effective in correcting for certain artifacts and drifts, its current implementation still leaves room for further improvement as the optical system already achieves shot-noise limited performance. One potential solution involves minimizing the time between internal and external self-referencing by generating alternating sample and reference droplets with the microfluidic chip.^37−40^ This method could significantly suppress all potential sources of short-term drift; but would require more complex chip designs and a different data read-out scheme. Nonetheless, variability between paired droplets—such as differences in size, position and refractive index—would ultimately limit the effectiveness of any referencing approach.

### Resolving Fast Dynamical Changes with ms-Resolution

**Figure 5:**
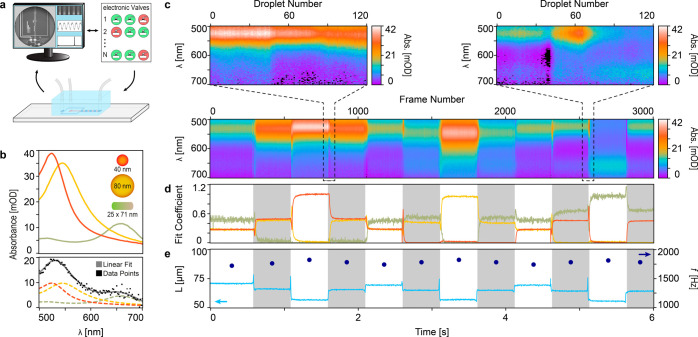
Measurement of fast spectral changes with 2 ms time resolution. (a) Schematic representation of experiment. (b) Top: AuNP component library obtained by averaging the absorbance spectra of individual AuNPs. Bottom: exemplary composition determination by fitting the read-out absorbance spectrum to a linear combination of the elements of the AuNP library. Dashed spectra correspond to the retrieved AuNP library components. Data points were acquired on a single droplet in the first valve state. (c) Time-resolved absorbance spectra measured for a predefined sequence of valve actuations modulating the sample composition. While single-droplet spectra are shown in the insets, the spectra in the main graph corresponds to the average of all drops within a frame and thus exhibits a higher SNR . (d) Corresponding time-resolved composition of the droplets expressed as the linear fit coefficient retrieved from the component library derived in (b). (e) Corresponding average droplet length per frame and droplet production frequency at a specific valve state. Alternating shaded regions in (d,e) represent the ground truth change in the valve state.

Leveraging the fast temporal resolution of the platform we validated its suitability to monitor fast dynamics using a model test system. Specifically, we used the platform to resolve rapid changes in a heterogeneous sample composition induced by microvalve state actuation. Three different AuNP species connected to different sample inlets—40 nm spheres, 80 nm spheres, and 25 nm × 71 nm rods—were used as the constituent components of the heterogeneous sample. To evaluate the sample composition at each time point, we first built a compound library from highly averaged droplet spectra of the individual AuNP samples (Figure 5b, top panel). This library was then used to fit a linear combination of the individual components and a flat offset to each spectrum previously extracted from the HSI stack. The offset accounted for changes in pressure attributed to the number of open inlet channels.

Said composition analysis can then be applied to each individual droplet in the experimental data set, as demonstrated for an exemplary droplet in Figure 5b (bottom panel) and the top panel of Figure 5c, enabling to follow rapid changes in sample composition with single-droplet resolution. To enhance the SNR, we averaged all droplets within a frame to generate a single spectrum, resulting in a 2 ms time-resolved absorbance spectral map (Figure 5c, bottom panel). Figure 5d shows the corresponding fit coefficients from each frame thereby resolving the sample composition in terms of the compound library. Based on these fit coefficients, the effective concentration or even the absolute number of particles of each component per droplet could be determined. There, rapid changes in sample composition followed by a quick stabilization fully match with the actuated changes in valve state and exhibit the characteristic time response signature associated with micro valve systems.^30^

Combining the information from the HSI and confocal channels we also time-resolved changes in droplet length and production frequency as a function of the actuated valve state (Figure 5e). Apart from a sharp peak after closing an inlet (short pulse of higher pressure due to the collapse of a flow channel) and a small dip upon opening an additional inlet (pressure release due to the opening of an additional channel) the droplet size stabilized almost immediately. With all three sample inlets open, the chip produced the largest droplets. Conversely, with only one aqueous inlet open, the chip produced the shortest droplets. These observations agree with the results obtained from the characterization of the droplet generation of the chip (Figure 2e), namely that the droplet production frequency is inversely proportional to the droplet length.

Although differences in valve types and geometries make it difficult to quantitatively compare the observed rise and fall times to other systems, these results prove that our platform can be applied to study fast dynamic systems. Specifically for this model system, detecting changes in sample composition was not limited by the spectral readout speed, but rather by the microfluidic valve actuation speed. Regarding the temporal resolution, averaging all droplets per HSI frame led to a 2 ms resolution; however, the time resolution per spectra increases to almost 500 μs if each droplet were considered individually at the expense of a lower SNR. Further resolution improvements—approaching 250 μs—can be achieved by increasing the droplet production frequency within the range accessible by the chip (Figure 2e), thus representing an almost 50-fold gain compared to similar work.^23^ Regarding the composition analysis, the lower nanorod OD in the compound library as well as a weaker SNR in the redder spectral region (Figure S8), led to higher noise levels in the fit coefficient with respect to the other AuNPs. Implementing approaches like single value decomposition^41^ or principal component analysis,^42^ could further improve the compositional analysis by deriving information from the spectral data set without prior knowledge of the compound library.

### DNA Sensing and Reaction Monitoring over Time

**Figure 6:**
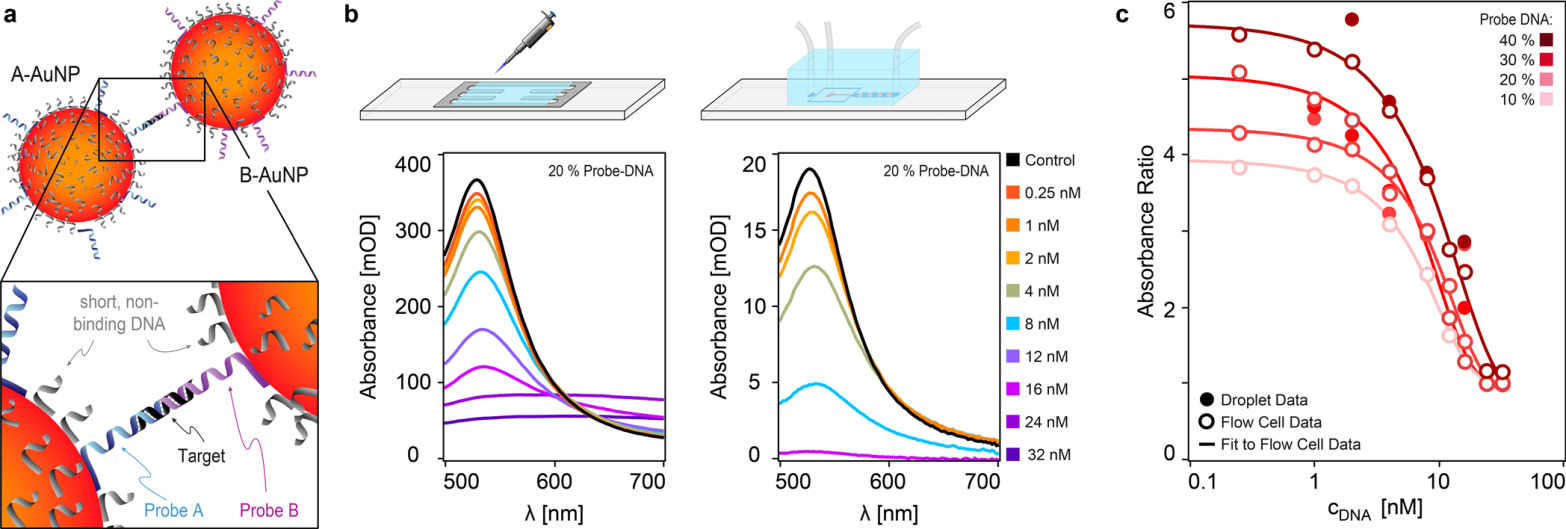
End-point sensing of DNA. (a) Schematic representation of the DNA-AuNP sensing system. (b) End-point validation of the DNA sensor for different target DNA concentrations measured in a flow cell and in a microfluidic droplet chip. (c) Corresponding dose–response curves from (b) for different probe DNA doping percentages.

To demonstrate the versatility of the platform as both a sensitive amplification- and label-free end-point and a real-time biosensor we showcase two proof-of-principle experiments using a DNA-AuNP system (Figure 6a). This system consists of two sets of AuNPs functionalized with different DNA capture probe strands (A- and B-AuNP) which in the presence of the target DNA strand hybridize to form dimers and larger agglomerates in a concentration dependent manner. Said agglomerations can then be measured by changes in the absorbance spectrum.

The first experiment leverages on the platform’s spectral sensitivity together with the DNA-AuNP sensor to detect a 22 nucleotide DNA sequence within pL droplets. As validation of the droplet platform, we first performed end-point dose–response measurements in a flow cell as a control, i.e. a system without droplets and a 25-fold larger OPL (Figure 6b). We then repeated these measurements in pL droplets. To account for possible nonspecific interactions between the sample, the presence of different interfaces, and to minimize artifacts associated with comparing absolute absorbance values we quantitated the dose response by a ratiometric metric. Namely, we compared the absorbance at two different wavelengths as λ_peak_/λ_610 nm_. Figure 6c shows the corresponding dose–response curves for the data recorded in flow cells (open circles) and in droplets (filled circles) for different doping percentages of the probe DNA on the functionalized AuNPs. The solid line represents a fit of the flow cell data points based on 4PL model (see Material and Methods and Supporting Information). The dose response curves for both flow cell and droplets show that the capture probe doping modifies the linear sensing range and sensitivity of the system. Although there are differences between flow cell and droplet data—expected due to the slight differences in the environment between the two systems—these results demonstrate that the DNA-AuNP system detects target DNA in the range between 250 pM and 32 nM, with a roughly 1 order of magnitude linear range. For comparison, Pei et al.^43^ observed DNA hybridization in a range between 0.5 nM and 10 nM with standard absorbance using a similar system (13 nm AuNPs, 30 nucleotide target); whereas Hwu et al.^44^ increased the linear sensing range to approximately 2 orders of magnitude by monitoring the dimerization of immobilized AuNPs via darkfield microscopy upon hybridization of the DNA target. Given a target length of only 22 nucleotides, our system provides a route for amplification-free high-throughput miRNA detection.^45−48^

**Figure 7:**
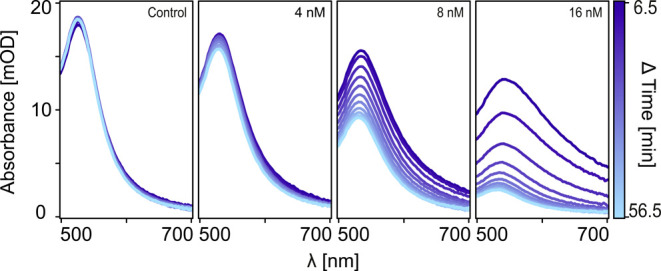
Multiplexed and long-term sample monitoring. Observation of slow dynamics caused by the formation of dimers and agglomerates of DNA-AuNPs for different concentrations of target DNA. Data were acquired 6.5 min after mixing DNA-AuNPs and target strands together. Sampling was performed every 5 min. More concentrations are displayed in Figure S9.

Using the same DNA-AuNP system, the second experiment exploited the measurement automation capabilities of the platform to monitor the evolution of slow dynamic processes in a multiplexed manner, over time scales ranging from minutes to hours. Specifically, we monitored the response of the DNA-AuNP sensor to five different target DNA concentrations and a negative control, measuring them sequentially every 5 min for up to an hour (Figures 7 and S9). The time-resolved spectra showed no changes for the negative control, while concentration-dependent changes were observed in the presence of the DNA, with rapid initial changes followed by a plateau after 50 min, indicative of the time scale for equilibration. Although performed on a model system, this second experiment highlights that our platform enables multiplexed real-time monitoring of dynamic processes in small volumes across different time scales.

## Conclusions

We presented an optofluidic platform that extracts content-rich information from single picolitre-sized droplets at high throughputs. The developed platform addresses a critical gap in high-throughput droplet analysis by integrating droplet microfluidics with HSI, self-referencing, and measurement automation. The platform features a highly controllable droplet-based microfluidic chip, a modular custom microscope optimized for broad-band absorbance measurements, but adaptable to fluorescence or Raman/SERS, and a robust data analysis workflow. All these features enable temporally stable and robust, self-referenced absorbance measurements for spectrally monitoring sample dynamics across various time scales, from ms to hours. We demonstrated such capabilities with a DNA-AuNP sensor system by performing both end-point measurements and long-term reaction dynamic monitoring.

In conclusion, our findings show that the developed optofluidic platform not only helps to close the current gap in high-throughput droplet analysis, but also significantly advances the potential for content-rich characterization, ultimately enhancing the scope and effectiveness of high-throughput screening applications. Because of the modular design of the platform, our approach can be extended to existing commercial microscopes by simply integrating the HSI module, at a cost that is competitive if not lower than high-speed spectrometers. We believe that our system will find widespread applications across various fields requiring fast, simple and cost-efficient screening of small sample volumes and large parameter spaces; or requiring long-term monitoring of dynamic systems with limited volume. For the latter we envision applications in real-time secretome monitoring of organ-on-a-chip, whereby our platform could continuously study the biochemical response to external stimuli of the organ-on-a-chip by sampling the supernatant containing the secretome through our chip. This application could have far-reaching implications in fundamental biomedical and pharmaceutical research, but also advance personalized medicine, drug development, and may even contribute to reducing the need for animal testing.

## Supporting Information Available

Reagents used, detailed description of mold- and chip fabrication, a precise description of the optical setup including all optical components, additional information concerning chip and hardware interfacing, setup calibration, data binning, and setup automation. Furthermore, it presents nine supplementary figures covering the microfluidic chip, hardware solutions to improve spectral quality as well as a comparison of externally self-referenced spectra vs uncorrected spectra, the calibration of the setup as well as the internal self-referencing process, a comparison of the platforms spectra to established standard methods, long-term absorbance measurements, a detailed noise analysis, and more data regarding long-term sample monitoring (PDF)
