# Supplementary material for: Hyperspectral Imaging for High Throughput Optical Spectroscopy of pL Droplets
Source: Anal Chem. 2025 Jan 29;97(5):2736–44. doi: 10.1021/acs.analchem.4c04731 (PMC11822737; doi:10.1021/acs.analchem.4c04731)
Supplement: Supplementary file 1 — ac4c04731_si_001.pdf [file ac4c04731_si_001.pdf]

SUPPORTING INFORMATION

Hyperspectral Imaging for High Throughput Optical Spectroscopy of pL Droplets

Marc Sulliger, Jaime Ortega Arroyo*, Romain Quidant*

Nanophotonic Systems Laboratory, Department of Mechanical and Process Engineering, ETH Zurich, 8092 Zurich, Switzerland.

Correspondence to: jarroyo@ethz.ch, rquidant@ethz.ch

Table of Contents:

Supporting Methods and Materials……pages S2 – S5
Supporting Figures…………………….…pages S5 – S9

SUPPORTING METHODS AND MATERIALS

Reagents

3M™ Novec™ 7500 fluorinated oil containing 2 % dSURF (DR-RE-SU-30) was purchased form Fluigent, France. 10x Tris Buffered Saline (TBS, 1706435) and 10% Tween 20 Solution (1610781) were purchased from Bio-Rad, USA and mixed to TBST (1x TBS containing 0.1 % Tween 20 Solution). 1,3-Bis(trifluoromethyl)-5-bromobenzene (290157-50g), ChemiBLOCKER (2170), phosphate buffered saline (PBS, P4474-1L), Tris Acetate-EDTA buffer (TAE, F4038-1L), sodium chloride (S9888-500G), and nuclease-free water (W4502-1L) were purchased from Merck, Switzerland. Tris Acetate-EDTA buffer was diluted in autoclaved water and mixed with sodium chloride to a 1.5x TAE, 1 M NaCl buffer.

Gold Nanoparticle

40 nm gold nanospheres, bare (citrate), 2.2 nM at 20 OD (AUCR40) and 80 nm gold nanospheres, bare (citrate), 230 pM at 20 OD (AUCR80) were purchased from nanoComposix, USA. Gold nanorods 25 nm x 71 nm, bare (citrate), 170 pM at 1.2 OD (A12-25-650-CIT-DIH-1-25) were purchased from Nanopartz, Canada.

Four Parameter Logistic Regression (4PL) Model

End-point DNA-AuNP data was fitted to the following four parameter logistic regression (4PL) model:

$$f([DNA]) = Absorbance\ Ratio = a_{sat} + \{(a_0 - a_{sat}) / (1+10^{([center] - [DNA])*p})\},$$

a_{sat} = top asymptote
a_0 = bottom asymptotes
[center] = target concentration at the center of the curve
[DNA] = concentration of the target
p = hill slope

DNA Strands

All oligos were purchased from Integrated DNA Technologies IDT, USA, and diluted to a stock concentration of 100 µM. The oligo sequences are as follows:

Oligo sequence	5’ → 3'
Probe strand A	CTG ATA AGC TAT TTT TTT TTT TTT TTT TTT TTT TTT TTT TTA AAA AAA AAA
Probe strand B	AAA AAA AAA ATT TTT TTT TTT TTT TTT TTT TTT TTT TTT TTC AAC ATC AGT
Short DNA	AAA AAT TTT T
Target	TAG CTT ATC AGA CTG ATG TTG A
Control	ACA GAT TGA GTT AGA CTT TCG T

Design of the Microfluidic Chip

The microfluidic chip is based on a two-layer architecture with microfluidic Quake valves in a push up configuration. The flow layer features a total of seven independently addressable aqueous/sample inlets as well as a waste outlet. All the sample inlets connect to a single channel before droplet generation. A flow focusing junction generates the droplets by combining oil and aqueous phases. The produced droplets flow into the outlet channel, designed to have a 5-fold lower hydrodynamic resistance than each of the inlet channels. Along the outlet channel, the droplets first pass through a short section composed of three meandric turns, which achieve reagent mixing by breaking the axial symmetry of the droplet internal flow patterns. The outlet channel then expands from 35 µm to 45 µm before reaching a 1 mm long channel section, here referred to as the measurement ROI. The control layer comprises nine independent inlets that overlap with specific areas of the flow channels, thereby forming the physical valves.

Fabrication of Molds for Microfluidic Chips

Individual molds for flow and control layer were fabricated by means of soft lithography using direct laser writing (µMLA, Heidelberg Instruments, Germany) on a 4-in silicon wafer (WSM4052525XB1314SNN1, MicroChemicals GmbH, Germany). The mold of the control layer was fabricated through a single exposure of 20 µm SU8 1060 negative photo resist (Gersteltec Engineering Solutions, Switzerland). The flow layer mold, on the other hand, required a two-step fabrication process to simultaneously allow for semi-rounded (valve areas) and squared (all other areas) channel cross sections. To achieve this, the wafer was first structured with SU8 1060 photo resist as described above. Then, the same wafer was coated again with 20 µm of AZ P4620 positive photo resist (Micro-Chemicals GmbH). Before structuring the photo resist by a second exposure, the design was aligned with the pre-existing SU8. A final baking step causes the AZ structures to slightly melt and reshape into semi-rounded cross sections whilst the SU8 photo resist undergoes a glass transition but maintains the squared channel profile. Finally, both molds were silanized with chlorothrimethylsilane (92360, Sigma Aldrich, Switzerland) at low pressure to improve longevity.

Fabrication of Microfluidic PDMS Chip

To produce the two-layer microfluidic droplet chips, the structures from the two molds were transferred to PDMS (Sylgard 184, Dow Chemical, USA) mixed at 10:1 w/w polymer to curing agent ratio. For the flow layer, the PDMS was drop-cast onto the respective mold to a height of approximately 5 mm. To make the control layer, a small amount of PDMS was spin-coated onto the mold at 2000 rpm resulting in a layer thickness of about 40 µm. Both molds were then placed under vacuum to degas until bubbles were no longer visible (typically around 60 min). Afterwards, the molds were baked in a convection oven at 80 °C for one hour. Once cured, the PDMS on the flow layer mold was peeled off the substrate, individual chips were cut out and inlet holes were punched. Then, the individual flow layer chips and the control layer mold (still covered with 40 µm of PDMS) were exposed to oxygen plasma (10s, 300 W, 8 sccm, Atto Low Pressure Plasm System, Diener Electronics, Germany) before manual alignment under a stereo macroscope (Z16 APO, Leica, Germany). For a stronger bond between the two layers, the aligned chips were baked again for 15 min at 80 °C in the oven. The chips were then cut off from the control mold and the inlet holes for the control layer were punched. To finish the assembly, the two-layer PDMS structure and microscope glass slides (17204894, Erpedia, USA) were exposed to oxygen plasma of the same conditions as before, bound together, and baked for another 15 min at 80 °C in the oven.

Flow Cell Fabrication

Flow cells were used as simple and fast alternative to droplet microfluidic chips to test the performance of the optical platform and to exclude droplet-specific contributions. Each flow cell consisted of twelve independent straight channels (width = 1.4 mm) open to atmospheric pressure on both ends. This design allowed for capillary-force driven loading of volumes down to 2 µL into each channel without any external equipment. The flow cells were fabricated from a microscope glass slide (17204894, Erpedia) and a cover glass (0101030, Paul Marienfeld GmbH & Co. KG, Germany) separated by a silicon spacer (500 µm, CWS-S-0.5, Grace Bio-Labs, USA). The assembly was solely based on surface energy. The channels were cut out of the silicon spacer by a cutting plotter (Cameo 4, Silhouette, USA).

Interfacing Microfluidic Chips with Lab Equipment

The sample inlets of the microfluidic chips were connected to Tygon® tubing (øin = 0.508 mm, øout = 1.524 mm, Saint-Gobain, France) via a bent metal pin (Tube AISI 304, 0.65 / 0.35 x 17.5 mm, Unimed SA, Switzerland). The tubing from the flow layer was connected to two types of sample reservoirs based on their size: i) for large volumes, 1.5 mL Eppendorf tubes interfaced with a P-Cap (P-CAP2-HP, Fluigent) or, ii) for volumes in the tens of µL, dispensing tips (LL ½" ID 0.34 mm, GONANO Dosiertechnik GmbH, Austria) with a Male Luer Lock to Barb Adapter (Darwin Microfluidics, France). Both types of reservoirs were connected to pressure controllers (LU-FEZ-2000 or LU-FEZ-7000, Fluigent) with 4 mm x 2.5 mm polyurethane tubing (917-2407, RS Pro, GB). The pressure controllers were operated both off-line, and programmatically via a computer by using a LineUP Link module (LU-LNK-002, Fluigent). The inlets to the control layer were connected to the same metal pins and liquid filled Tygon® tubing as described above. The tubing from the control layer was then interfaced to a custom-built electronic valve unit that was programmatically controlled by a computer.

Passivation of Microfluidic Tubing

Passivation of the microfluidic Tygon® tubing - if performed - was achieved by first rinsing the tubing with a 10-fold dilution of ChemiBLOCKER in TBST. Then, the tubing was incubated with the same reagent for a few minutes before being thoroughly rinsed with PBS. Finally, the tubing was flushed with nitrogen for a few seconds.

Microscope

The custom-built microscope follows a flexible and modular approach combining three optical paths:

(i) The HSI path is based on a transmission microscope. For illumination a 554 nm fiber coupled light emitting diode (MINTF4, Thorlabs, USA) was connected to a 550 µm multimode fiber (M37L02, Thorlabs). The output of the fiber was imaged onto the sample by a telescope comprising a f = 11 mm aspheric lens (C397TMD-A, Thorlabs) and a f = 40 mm achromatic doublet lens (AC254-040-A, Thorlabs) arranged in a 4f configuration. The sample was positioned onto a custom built sample holder built on top of xyz-piezo translation stage (nanoCube, Physik Instrumente GmbH, Germany) mounted on top of a manual xyz-translations stage with a range of 25 mm x 25 mm x 12.5 mm. Light from the sample was collected by an 4x, 0.2 NA objective (TL4S-SAP, Thorlabs) before passing through a dichroic mirror (long pass 490 nm, DMLP490L, Thorlabs) and imaged onto a slit (width = 50 µm, VA100/M, Thorlabs) to produce a quasi 1D image. A second f=150 mm achromatic doublet lens (AC508-150A, Thorlabs) relay imaged the BFP of the objective onto two subsequent but counter-rotated Amici prisms (117240, Equascience, France) to spectrally disperse the light perpendicular to orientation of the slit. To allow for a multichannel readout a 90:10 (R:T) beam splitter (BSX16, Thorlabs) was introduced before the Amici prisms. Finally, the light reflected from the beamsplitter and dispersed by the prisms was imaged onto a CMOS camera (GS3-U3-23S6H, Sony IMY174 Sensor, 1200 x 1920 pixel, 5.86 µm x 5.86 µm pixel size, Point Grey, FLIR Systems, USA) by a f = 200 mm achromatic doublet lens (AC508-200-A, Thorlabs). This channel provided an effective 4x magnification (i.e. 1.488 µm per pixel) and an average spectral dispersion of 0.782 nm / pixel between 504 - 613 nm.

(ii) The confocal detection path corresponds to the portion of light transmitted through the above-described 90:10 beamsplitter. This light was focused using a f = 150 mm achromatic lens (LA1433-A, Thorlabs) onto an APD (APD120A2/M, Thorlabs) with a 50 µm pinhole (P50K, Thorlabs) mounted right in front of it. All optical elements were arranged in a 4f configuration with respect to the slit, leading to an effective 3x magnification.

(iii) The big FoV path corresponds to a spatially incoherent digital holographic optical system which was based on a common-path microscope operating in reflection with all optical elements arranged in a 4f configuration. For the illumination, a 415 nm fiber coupled light emitting diode (M415F3, Thorlabs) was connected to a 600 µm multimode fiber (M29L02, Thorlabs). The light out-coupled from the fiber by a f = 6.2 mm aspheric lens (C171TMD-A, Thorlabs) passed through a 1:1 relay-imaging system formed by two f = 125 mm achromatic doublet lenses (AC254-125-A, Thorlabs). In between the relay imaging system, a 50:50 beamsplitter (BSW27, Thorlabs) was placed to separate the illumination and imaging paths. This module was coupled to the rest of the optical microscope via a dichroic mirror (long pass 490 nm, DMLP490L, Thorlabs). Light reflected off from the dichroic mirror was focused onto the sample using the same 4x, 0.2 NA objective (TL4S-SAP, Thorlabs) as before. In the imaging arm, light from the sample was collected by the same objective and was subsequently imaged onto a CMOS camera (a2A4504-18umPRO – Basler ace 2, 4504 x 4504 pixel, 2.74 µm x 2.74 µm pixel size, Basler AG, Germany) upon reflecting off the 50:50 beamsplitter.

Calibration of Hyperspectral Camera (Mapping Pixels to Wavelength)

The HSI path of the system provides images with both spatial (y-axis) and spectral (x-axis) information. However, the information is reported according to the reference system of the camera: pixels. To read-out spectral information, a coordinate transformation from pixels to wavelength is required. We achieved this by a calibration step, detailed in Figure S4, which used a total of nine filters (FF02-472/30-25, Semrock, USA, and MF497-16, FL532-10, FB570-10, FLH635-10, FB650-10, FB700-10, FL740-10, FGB67, Thorlabs) with known and sharp absorption peaks. Said filters were mounted in front of the light source (before the sample stage) and imaged along the HSI path and compared against a reference spectrometer (USB4000, Ocean Optics, Ocean Insight, USA). The recorded data was then temporally averaged (2000 frames recorded over 40 s for the hyperspectral images, and 100 scans for the reference spectrometer) to increase the signal to noise ratio. For each filter, the wavelength of the absorbance peak was found in the spectrometer data. In the HIS channel, the pixel position of the absorbance peak along the spectral axis (x-axis) was located along each spatial coordinate (y-axis). Finally, the detected pixel-positions were mapped to the corresponding wavelengths and fit to a 3rd order polynomial. The resulting fit coefficients were then used, to transform the coordinates of the raw hyperspectral images. Note that by doing this, we automatically accounted for smile-aberrations.

Spectral Window and Data Binning

In the current implementation, the platform reads spectra between 490-700 nm. The lower and upper limit were set by the long pass 490 nm dichroic mirror in the optical setup, and the spectra of the light source itself, respectively. Previous tests have shown that for a shot noise limited and fixed photon budget system, the SNR for reading out spectral features does not improve when sampling beyond the Nyquist criteria. Therefore, we chose a spectral resolution of 2 nm by pixel binning along the spectral axis of the transformed HSI data.

Automated Measurements based on .txt File Sequences

Our microfluidic chips were equipped with micro-valves able to close flow channels pressurized up to 80 % of the applied pressure to the control layer. Thus, a fully pressurized microfluidic chip could be operated solely based on actuating the required microvalves which means that the entire microfluidic operation of the system could be programmatically controlled. To do so, we implemented a producer/consumer queue structure in LabView (2020, National Instruments, USA) to perform a set of specific tasks based on the four commands “open channel x”, “close channel x”, “wait” and “measure”. These tasks were then pre-programed into a .txt- file which then could be uploaded and sent to the electronic valves and camera respectively. A typical measurement of a single sample would thereby look as follows and usually last between 20 and 30 seconds: “open channel x” > “open channel waste” > “wait” > “close channel waste” > "open channel main” > “wait” > “measure” > “close channel main” > “close channel x” > “open channel matrix” > “open channel waste” > “wait” > “close channel waste” > "open channel main” > “wait” > “measure” > “close channel main” > “close channel matrix”.

SUPPORTING FIGURES

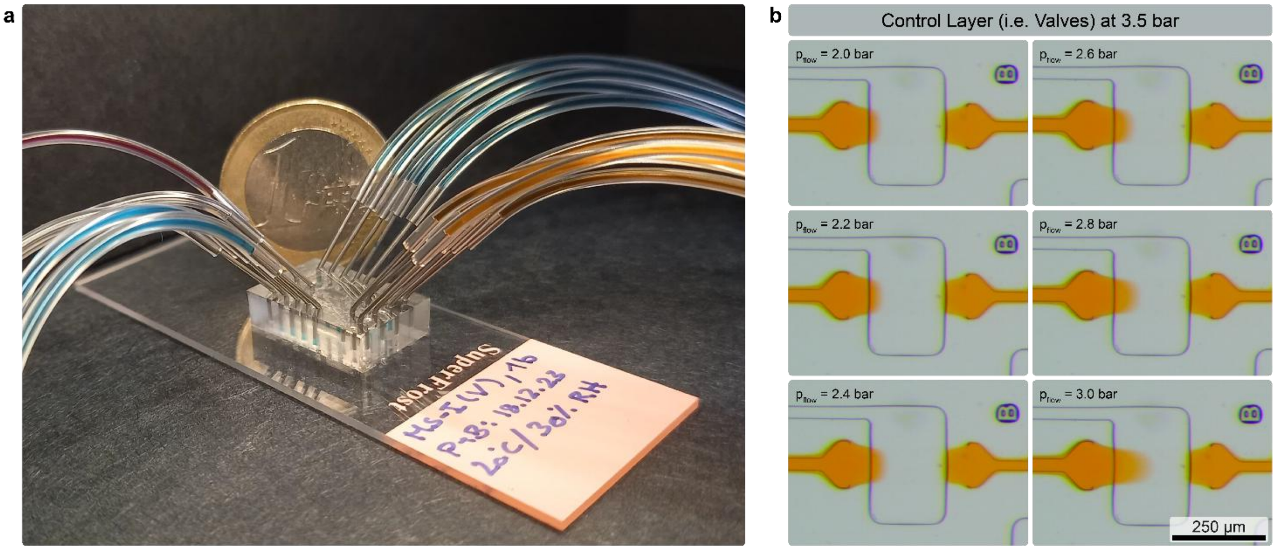

Figure S1. Assembled microfluidic chip and push-up valve characterization. (a) Image of an assembled microfluidic chip in comparison to the size of a 1 Euro coin. Orange and purple inlets connect to the flow layer (aqueous and oil inlet, respectively). Blue labeled tubes pressurize the control layer. The two transparent tubes (in the back to the left) connect to the two outlets. (b) Microscopy images of a closed push-up valve under 3.5 bars of pressure for different pressure conditions of the flow channel (orange). All information relevant to the characterization are presented in the figure.

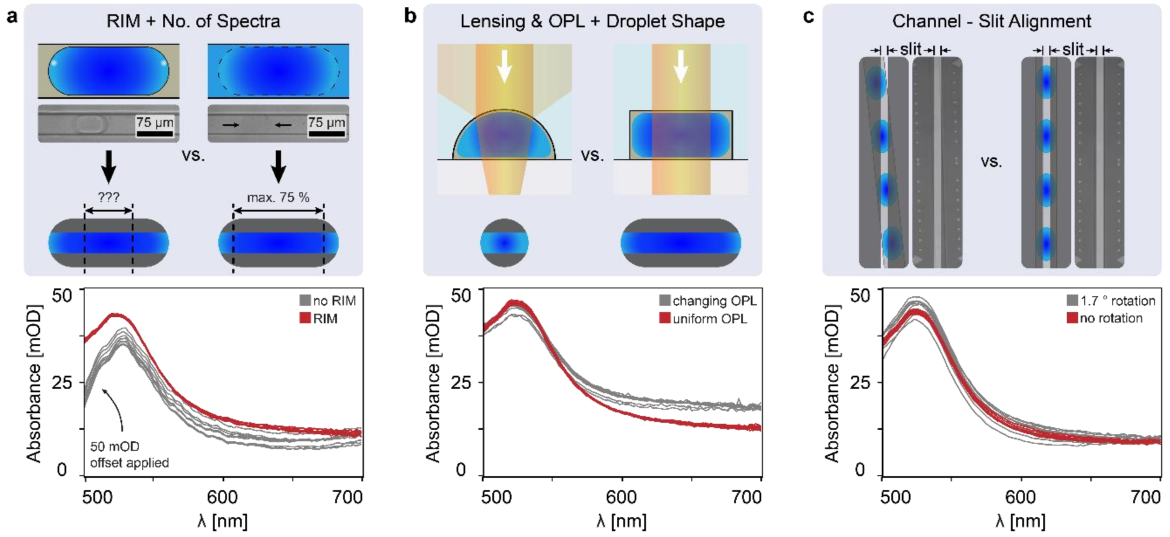

Figure S2. Important aspects when working with droplets: hardware solutions. (a) Refractive index matching adjusts the refractive index of the oil phase to match the one of the dispersed phase and suppresses scattering. For plug-like droplets, it then allows to use up to 75% of the droplets length for spectral read out. (b) A rectangular channel cross-section in combination with plug-like droplets allows for a uniform optical path length and avoids lensing effects. (c) A precise channel slit alignment guarantees that each position recorded in HSI measures a similar cross-section of the microfluidic chip. Each spectrum is an average of 100 droplets recorded at equally spaced positions along the channel. No external self-referencing has been applied.

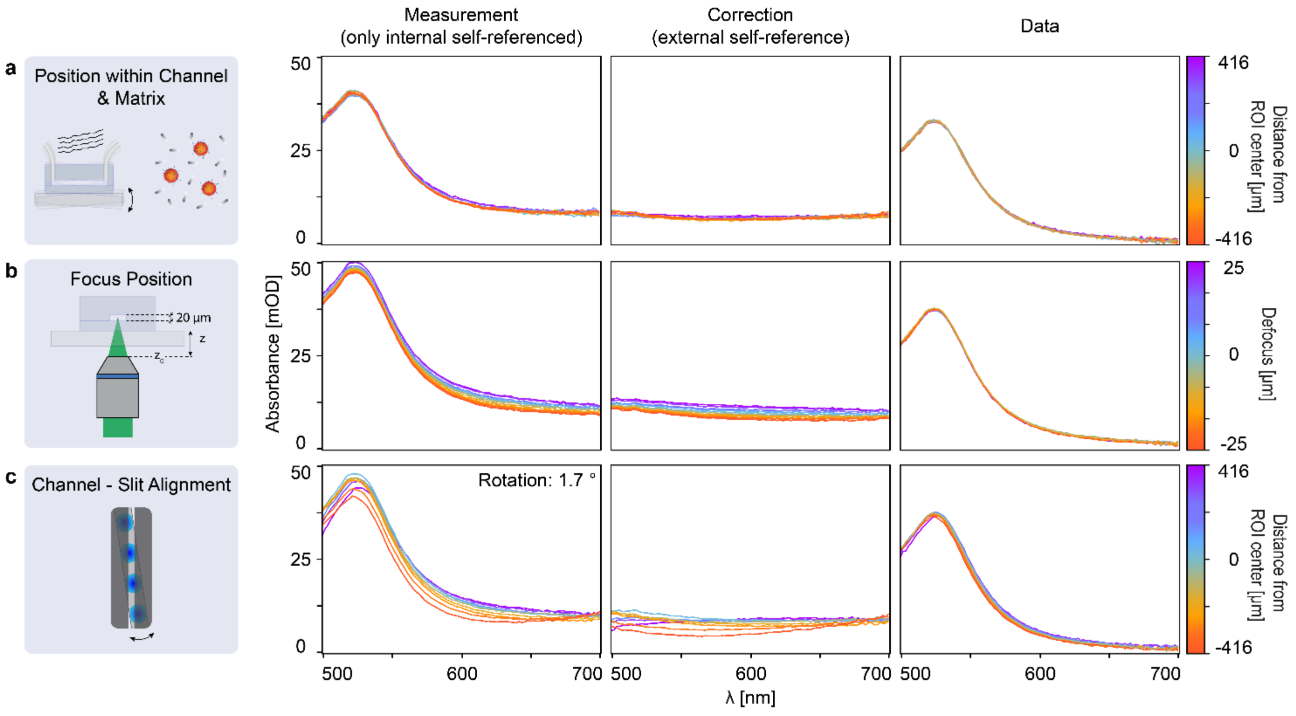

Figure S3. Correction due to external self-referencing. Applying external self-referencing allows to retrieve data even from distorted measurements. (a) For an otherwise unaffected measurement, effects related to the position within the channel (i.e. channel defects, static imprints, tilts in wavefront, etc.) and the matrix of the sample are corrected. (b) A change in focus position even over a range of 50 µm (note, that the channel height is only 20 µm) can be accounted for. (c) Finally, tilts in the channel-slit alignment can be addressed as well.

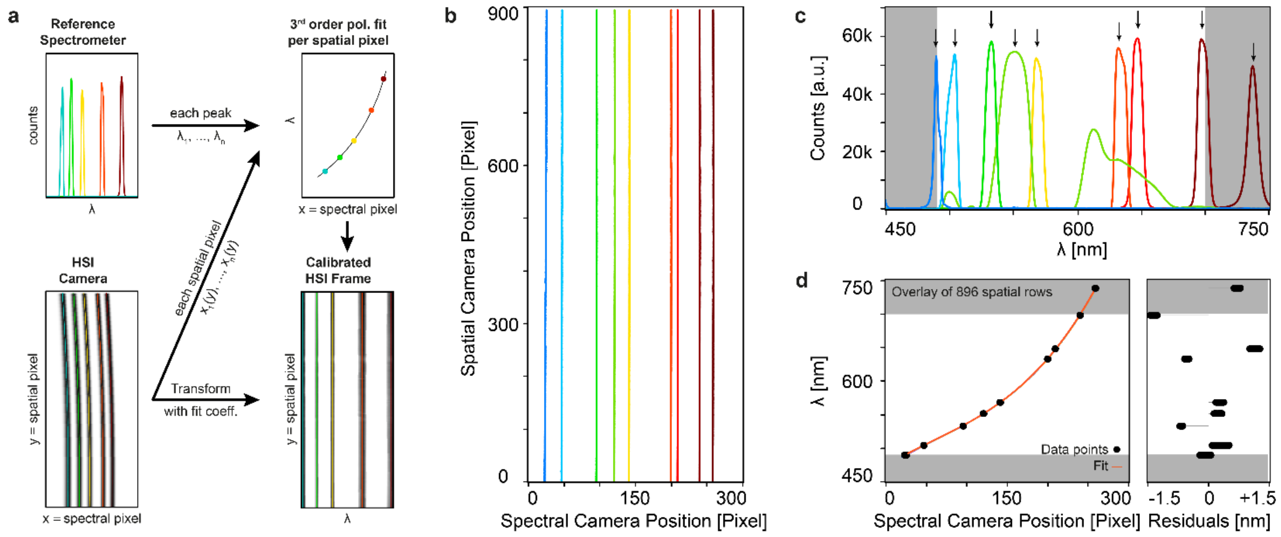

Figure S4. Calibration of hyperspectral camera. (a) Schematic representation of the calibration process. In short: Spectral filters with known peaks are measured with a reference spectrometer to identify the wavelength of the filter peak. At the same time, the filters are measured with the HSI setup. The maximum position of each filter peak in the HSI image is determined for each spatial position (y-axis). Then, the wavelength recorded from the reference spectrometer is mapped to the peak positions recorded with the HSI setup for each spatial position. The data points are fitted to a 3rd order polynomial for each spatial position. Using the fit coefficients, the spectral axis (x-axis) of the initially recorded HSI image is transformed from a pixel to a wavelength reference system. Note, that with this transformation, one automatically corrects for smile aberrations. (b) Recorded peak positions based on the HSI camera. Note, that the extent of smile is less than 4 pixels over the full 896 spatial pixels. (c) Recorded spectral peaks using a reference spectrometer. The used peaks are indicated with arrows. (d) Overlay of the fit of all 896 spatial pixel positions with the mapped data points (left panel) and corresponding residuals per data point (right panel). The white areas in (c) and (d) are actually used for spectral read-out whilst the grey areas are only included to ensure a smooth calibration even at the edge of the spectral window. Note, that one could easily extend the number of calibration points by adding more spectral filters or a calibration lamp.

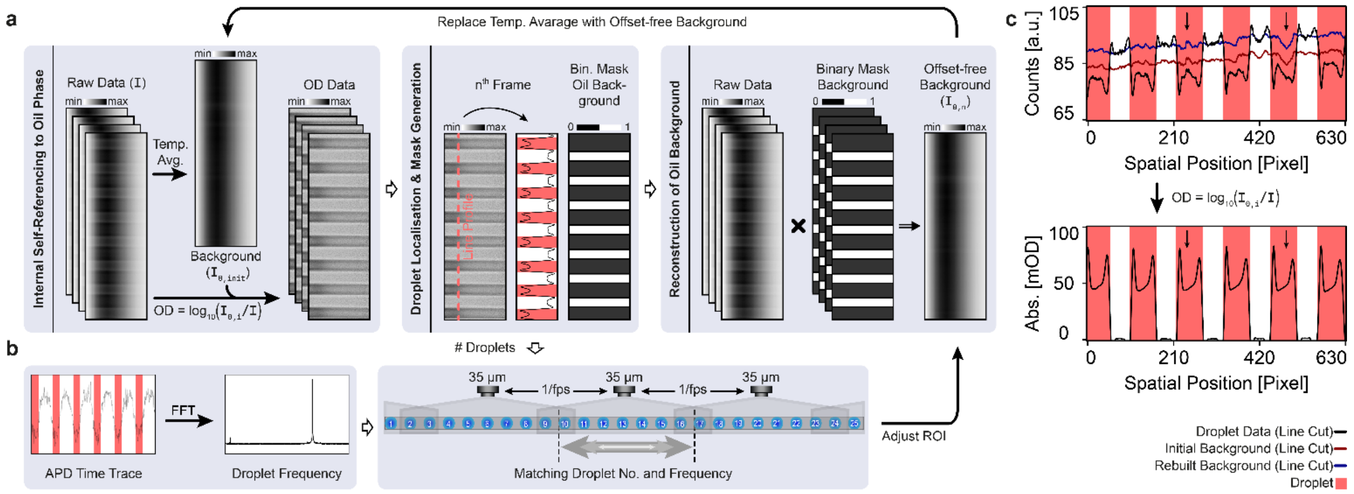

Figure S5. Background extraction workflow and frequency matching. (a) Schematic description of the extraction workflow. In short: OD data are extracted by iteratively improving the initially estimated background based on a time average over the entire HSI stack. Then, the droplets are localized by means of a line-profile along the spatial axis of the hyperspectral image and a mask for the background is generated. The mask stack is multiplied with the initial HSI stack to solely extract background regions from the HSI frame and reduced to a single frame. The latter represents an offset-free background and is used in a 2nd (and potentially 3rd) iteration to read out properly internally self-referenced OD data. (b) Based on the APD time trace and hence the droplet production frequency, the ROI is spatially adjusted to match the droplet production rate with the number of droplets that have been read out. This is purely based on post-processing and only necessary when each droplet needs to be considered individually. (c) Illustration of the difference between the initial background and the rebuilt background and the OD data generated from it. Note that the internal self-referencing takes care of all static background features (see arrows) and the overall tilt in the illumination profile.

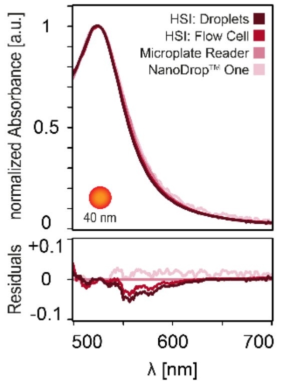

Figure S6. Comparison of HSI-based spectra of 40 nm AuNPs with standard methods. Normalized data for droplet and non-droplet-based measurements. Droplet and flow cell data were measured using the described optical setup and microfluidic chips. Thereby, the flow cell measurement excludes all effects specific to droplets. The latter were produced with 1270 Hz and had a length of 95.8 µm. For the reference measurement a microplate reader (Synergy H1, BioTek; V = 200 µL) and a NanoDropTM One (ThermoFisher Scientific; V = 2 µL) were used. For all samples, the background was accounted for by a matrix (i.e. buffer) measurement. The residuals compare all measurements to one another using the microplate reader as the ground truth. Note, the largest deviation of the droplets and flow cell data are located in the region with fewest calibration points (Fig. S2).

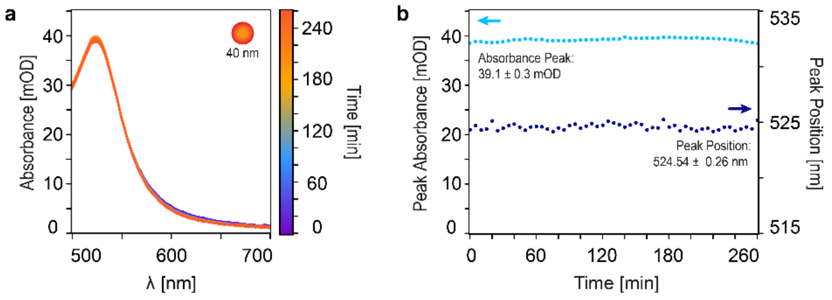

Figure S7. Long-term stability measurement of 40 nm AuNPs over 4 h and 20 min. (a) Spectra of 40 nm AuNPs recorded every 5 minutes and produced at a frequency of 1.36 ± 0.04 kHz and a length of 94.6 ± 0.9 µm. Each curve is an average of 1000 droplets. (b) Tracking of the peak absorbance and peak position. The indicated average and standard deviation thereby account not only for the stability of the setup but also reflect the fluctuations in the sample composition. In fact, one could use our system to observe changes related to sedimentation and agglomeration of particles that take place over large time scales.

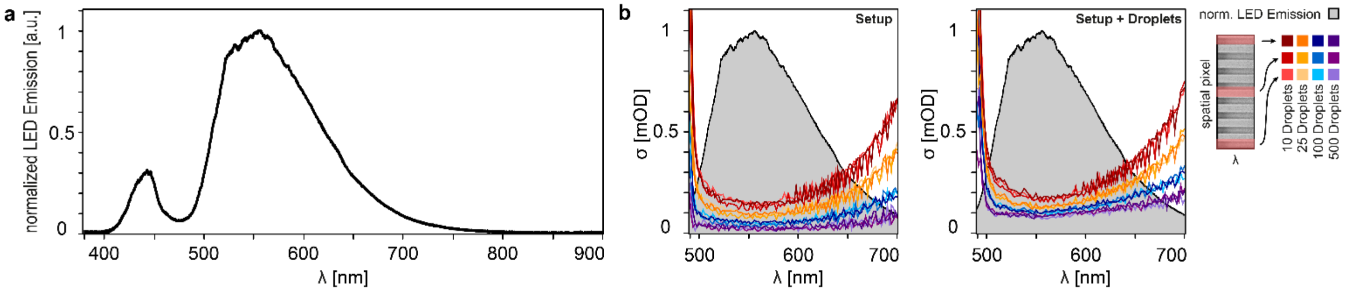

Figure S8. LED emission spectrum and spectral noise assessment. (a) Normalized emission spectrum of the fiber coupled MINTF4 LED. (b) Spectral noise assessment for the HSI setup considering contributions from the just the setup (left panel) and contributions from both the setup and droplet (middle panel) analyzed at three different spatial positions of the ROI. Right panel: cartoon depicting the spatial regions from which the spectral noise was analyzed (red area). Each spatial region corresponding to the beginning, centre and end of the ROI comprises a width of 104.1 µm. This figure is based on the same data set as Fig. 4c in the main manuscript. Note that the different curves (for the setup itself) are shot noise limited with the lowest value matching the maximum of the illumination spectrum.

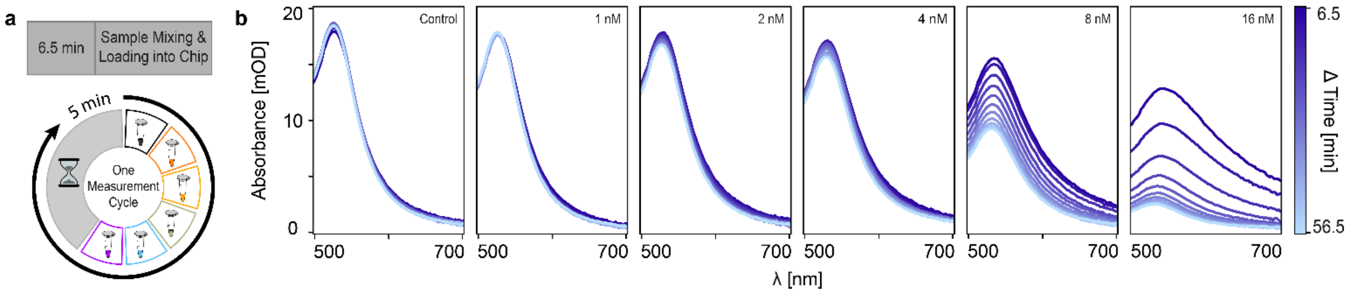

Figure S9. Multiplexed and long-term sample monitoring. (a) Schematic representation of automated measurement procedure. Data acquisition started 6.5 min after mixing DNA-AuNPs and target strands together and was performed sequentially every 5 min. (b) Observation of slow dynamics caused by the formation of dimers and agglomerates of DNA-AuNPs for different concentrations of target DNA concentrations.
